# Rapid Synthesis of Silver Nanoparticles from *Fusarium oxysporum* by Optimizing Physicocultural Conditions

**DOI:** 10.1155/2013/796018

**Published:** 2013-10-10

**Authors:** Sonal S. Birla, Swapnil C. Gaikwad, Aniket K. Gade, Mahendra K. Rai

**Affiliations:** ^1^Department of Biotechnology, Sant Gadge Baba Amravati University, Amravati, Maharashtra 444 602, India; ^2^Department of Biology, Utah State University, Logan, UT 84322, USA; ^3^Visiting Scientist, Institute of Chemistry, Biological Chemitry Labotrratory, Universidade Estadual de Campinas, Campinas, SP, Brazil

## Abstract

Synthesis of silver nanoparticles (SNPs) by fungi is emerging as an important branch of nanotechnology due to its ecofriendly, safe, and cost-effective nature. In order to increase the yield of biosynthesized SNPs of desired shape and size, it is necessary to control the cultural and physical parameters during the synthesis. We report optimum synthesis of SNPs on malt extract glucose yeast extract peptone (MGYP) medium at pH 9–11, 40–60°C, and 190.7 Lux and in sun light. The salt concentrations, volume of filtrate and biomass quantity were found to be directly proportional to the yield. The optimized conditions for the stable and rapid synthesis will help in large scale synthesis of monodispersed SNPs. The main aim of the present study was to optimize different media, temperature, pH, light intensity, salt concentration, volume of filtrate, and biomass quantity for the synthesis of SNPs by *Fusarium oxysporum*.

## 1. Introduction

Nanotechnology is mainly concerned with the synthesis of nanomaterials using different systems and their applications. At nanoscale level, materials have different chemical, physical, optical, magnetic, and electrical properties due to their large surface area to volume ratio. 

One of the most important aspects of nanotechnology is synthesis of nanoparticles (NPs) (one dimension less than 100 nm), which form the core part of the nanomaterials. Nanoparticles possess more surface atoms than micro-particles, which enhance their functional capabilities. Now a days, NPs influence various sectors in the areas of agriculture [1], environment, health care, and consumer goods [2]. They are also used for their unique properties in biosensing, photonics, and electronics [3]. The importance of biological synthesis has been realized globally because chemical methods are capital intensive and toxic. Thus, the need for clean, ecofriendly, cost-effective, and biocompatible synthesis of metal nanoparticles encouraged the researchers to exploit the biological sources as nanofactories [4–6]. It was found that fungi score more advantages over other biological systems because of their high tolerance towards the heavy metals [7]. Rai and coworkers [8] proposed the term “Myconanotechnology” for the synthesis of nanoparticles by using fungi. Fungi have the potential to provide relatively quick and ecologically “clean” metallic nanoparticles. There are many reports on the mycosynthesis of silver NPs (SNPs) [9, 10]. Application of fungi for production of SNPs is potentially exciting because of their ability to secrete large amount of proteins [11].

Among various metals, silver has been in use since antiquity in the form of metallic silver, silver nitrate, and silver sulfadiazine. But due to the arrival of several antibiotics, the use of these silver compounds has been weakened remarkably. Use of silver has been again realized in the form of SNPs, which show the significant antimicrobial activity against the multidrug resistant microorganisms owing to their reduced size [4]. Due to antimicrobial activity, SNPs are extensively used in dental materials [12], coating stainless steel in medical devices [13], cosmetics [14], and water treatment [15]. Antibacterial activity of SNPs was studied by various groups [16, 17]. Due to vast emerging applications of SNPs in distinct fields, there is increase in demand for SNPs, and to fulfill the demand, there is a pressing need to increase their yield, for which optimization of the process is very important step. A little contribution has been made regarding the effect of cultural and physical conditions on the biosynthesis of SNPs. The synthesis of SNPs at nanorange is still a challenge. In order to increase the yield and the shelf-life (stability) of SNPs with minimum investment, it is necessary to optimize the cultural conditions and various physical parameters like pH, light intensity, and temperature. To our knowledge, there is no prior report on the optimization of all these conditions for large scale synthesis of SNPs. 

We report the optimization of different media, temperature, pH, light intensity, salt concentration, volume of filtrate and biomass quantity for mycosynthesis of SNPs by *Fusarium oxysporum*. 

## 2. Experimental

### 2.1. Isolation and Identification of Test Fungus

The test fungus was isolated from decayed banana fruit on potato dextrose agar and incubated at 28°C for 4-5 days. Individual fungal colonies were picked and further purified by subculturing on potato dextrose agar medium. 

The fungus was identified by cultural (texture of mycelia, colony colour) and microscopic characteristics (macro- and microconidia and chlamydospores) by using Seifert's key [18] and Leslie's Laboratory Manual [19]. Further identity of fungus was confirmed by nuclear ribosomal DNA internal transcribed spacer (ITS) sequencing. For this fungal, genomic DNA was isolated by using Fungal gDNA extraction kit (Chromous Biotech Pvt. Ltd. Bangalore, India). ITS region was amplified by ITS1 5′-TCCGTTGGTGAACCAGCGG-3′ and ITS4 5′-TCCTCCGCTTATTGATATGC-3′ primer. Polymerase chain reaction amplified regions were sequenced. The sequence was used for the basic local alignment search tool analysis at National Centre of Biological Information (NCBI) server for the homology searching. 

### 2.2. Extracellular Synthesis of Silver Nanoparticles

The mycelia of *F. oxysporum* were inoculated in 250 mL Erlenmeyer flasks, each containing 100 mL of potato dextrose broth (PDB) medium and incubated at 25 ± 2°C for 5 days. Later, mycelia were harvested by filtration through Whatman filter paper no. 42 and washed thrice with sterilized distilled water to remove the traces of medium on fungal biomass. The washed mycelia were resuspended into 100 mL sterilized distilled water and incubated at 25°C for 24 hrs. Again, mycelia were harvested by filtration through Whatman filter paper no. 42. Then, cell filtrate was treated with 1 mM silver nitrate solution and incubated at room temperature. Positive controls containing cell free filtrate without silver nitrate and only 1 mM silver nitrate as negative control were also maintained.

### 2.3. Characterization of Silver Nanoparticles

The detection of SNPs was primarily carried out by visual observation of colour change of the fungal filtrate after treatment with silver nitrate. Appearance of dark brown colour of fungal cell filtrate indicates the formation of SNPs. Further, SNPs were characterized with the help of dual beam UV-Visible spectrophotometer (Shimadzu-UV 1700) by scanning the absorbance spectra in 200–800 nm range of wavelength. It is well known that, for monodispersed nanoparticles, only one plasma band is obtained and the increase of its intensity is an indication of the reaction advance degree with subsequent increment in the number of particles.

#### 2.3.1. Characterization of Silver Nanoparticles by Nanoparticle Tracking and Analysis (NTA)

The particle size and distribution was executed by Nanoparticles Tracking and Analysis (NTA) with LM-20 (NanoSight Ltd. UK). The size distribution of nanoparticles, which can be obtained on a particle-by-particle basis by LM-20, was studied. NTA enables separation of particles population by size and intensity, microscopically visualizing individual nanoparticles in suspension and simultaneously determining their Brownian motion. The NTA calculates the particles size by distance travelled by them. Size calculation was based on Stokes-Einstein equation, applied to particles with its size. For each distribution, data are given as mean (the average particles size measured) and mode (most frequent particle size found) terms.

#### 2.3.2. Characterization of SNPs by Fourier Transform Infrared Spectroscopy (FTIR)

FTIR analysis of the dried powder of SNPs was carried out by scanning the spectrum in the range 400–4,000 cm^−1^ at a resolution of 4 cm^−1^ (PerkinElmer 1600 instrument, USA). FTIR measurements were made to locate the possible biomolecules, which are responsible for the reduction of silver ions to SNPs and stabilization of SNPs in colloidal solution. To prepare dried powder of SNPs and to remove other biomolecules present in broth, the fungal treated broth was centrifuged at 12000 g for 15 minutes. Supernatants were discarded, and pellets of SNPs were washed three times with autoclaved distilled water. The dried powder of SNPs was subjected to FTIR analysis.

#### 2.3.3. Characterization of SNPs by Transmission Electron Microscopy (TEM)

To understand the morphology of SNPs synthesized by applying all optimized conditions and without optimized conditions, the transmission electron microscopic analysis was performed. For TEM measurements, a drop of solution containing synthesized SNPs was placed on the carbon coated copper grids and kept in infrared light until sample gets dried before loading them onto a specimen holder. TEM micrographs were taken by analysing the prepared grids on Philips CM 200 super twin's TEM instrument operating at 200 kV (0.23 nm resolution). The crystalline nature of metallic SNPs was confirmed by selected area diffraction pattern.

### 2.4. Optimization of Conditions

For the large scale and stable mycosynthesis of SNPs, it is necessary to optimize the physical and cultural conditions. Several experiments were carried out concerning the rate of synthesis and stability of SNPs. The parameters such as media, pH, temperature, light intensity, quantity of biomass, concentration of silver nitrate, volume of filtrate and time of reaction were standardized for the rapid and maximum synthesis of SNPs. For each condition, there was respective control. All experiments were performed in triplicate.

#### 2.4.1. Effect of Different Media

Effect of ten different media, namely, Czapek broth (sucrose 0.3 g, NaNO_3_ 0.03 g, K_2_HPO_4_ 0.01 g, MgSO_4_ 0.005 g, KCl 0.005 g, and FeSO_4_ 0.0001 g 100 mL^−1^ pH 7.3),  glucose peptone yeast broth (glucose 5 g, peptone 1 g, yeast extract 1 g), gluten glucose broth, lipase assay medium (peptone 3 g, NaH_2_PO_4_ 1.2 g, KH_2_PO_4_ 0.2 g, MgSO_4_·7H_2_O 0.03 g, CaCl_2_ 0.025 g, and olive oil 1 g, pH 6.5), malt glucose yeast peptone broth (malt extract 0.3 g, yeast extract 0.3 g, peptone 0.5 g, and glucose 1 g, pH 7), potato dextrose broth (potato 20 g and dextrose 2 g, pH 6.4), protease production medium (glucose 0.2 g, casein 0.05 g, peptone 0.05 g, yeast extract 0.05 g, KH_2_PO_4_ 1 g, MgSO_4_ 0.5 g, and FeSO_4_ 0.01 g, pH 8.4), Richard's broth (sucrose 0.5 g, KNO_3_ 0.1 g, KH_2_PO_4_ 0.025 g, and MgSO_4_·7H_2_O 0.025 g), Sabouraud broth (dextrose 4 g and peptone 1 g), and sucrose peptone yeast broth (sucrose 5 g, peptone 0.1 g, and yeast extract 0.1 g, pH 6.5), was studied on the formation of SNPs. All the media were screened for the optimum and stable nanoparticles synthesis. The fungal mycelia were grown for 7 days in 250 mL Erlenmeyer flasks, each containing 100 mL of test medium. The synthesis of SNPs was carried out as mentioned above in Section 2.2.

#### 2.4.2. Effect of Different Quantity of Biomass

In order to study the effect of biomass quantity on synthesis of SNPs, 0.2 g, 0.4 g, 0.6 g, 0.8 g, 1.0 g, 2.0 g, 3.0 g, 4.0 g, 5.0 g, and 6.0 g mycelia of previously grown culture on MGYP medium were suspended in distilled water to optimize the quantity required for the optimum synthesis of SNPs. The SNPs were synthesized as mentioned above in Section 2.2.

#### 2.4.3. Effect of Different Hydrogen Ion Concentrations

Better synthesis and stability of SNPs were studied by suspending biomass in distilled water having different pH, namely, pH 3, pH 5, pH 7, pH 9, and pH 11, and pH of the filtrate was also maintained with 1 N HCl and 1 M NaOH.

#### 2.4.4. Effect of Different Temperatures

Effect of different temperatures on the rate of synthesis of SNPs was studied in two ways. First, fungal biomass was suspended in distilled water and exposed to different temperatures ranging between 0°C, 20°C, 40°C, 60°C, 80°C, and 100°C for overnight to increase the extracellular protein secretion by giving heat shock. In second, fungal filtrate after treating with 1 mM silver nitrate was kept at different temperatures ranging between 0°C, 20°C, 40°C, 60°C, 80°C, and 100°C till the synthesis of SNP was observed.

#### 2.4.5. Effect of Different Light Intensities

Effect of different light intensities of different light sources, like 15.5 (15 Watt light), 93.1 (U.V. light), 141.3 (40 Watt yellow light), and 190.7 (40 Watt fluorescence light) lux on the rate of nanoparticles synthesis, was studied. The SNPs were also synthesized in sunlight, that is, 750 × 100 lux, and in dark. Light intensity was measured using Lux meter (TES-1332 digital Lux meter).

#### 2.4.6. Effect of Different Concentrations of Silver Nitrate and Filtrate Volume

In order to increase the synthesis of the SNPs, effect of different concentration of silver nitrate 0.1 mM, 0.2 mM, 0.3 mM up to 2 mM and effect of filtrate volume (at fixed concentrations of 1 mM AgNO_3_) were studied. Time course of SNPs synthesis was studied by measuring UV-Vis spectra after regular intervals of 5 min up to 45 min.

## 3. Results

### 3.1. Cultural and Molecular Characterization of the Fungus

The colony grew with aerial cottony mycelia and violet pigmentation on potato dextrose agar medium. Macroconidia were slightly curved and thin-walled. Basal cells were pointed with three septa. Kidney-shaped microconidia were abundantly produced in mycelia. Chlamydospores formed in short chain with rough wall. On the basis of all these cultural and microscopic characteristics, the fungus was identified as *Fusarium oxysporum*. Further, the identity was confirmed by ITS-rDNA sequence comparison with the available sequences in the data base (NCBI) using online BLAST analysis programme. Amplified sequence entered into the BLAST algorithm of the NCBI data base showed 99% similarity to available data of *F. oxysporum.* The amplified sequence was deposited in EMBL GenBank (Accession no. FR 851229).

### 3.2. Characterization of Silver Nanoparticles by UV-Visible Spectroscopy and NTA

**Figure 1:**
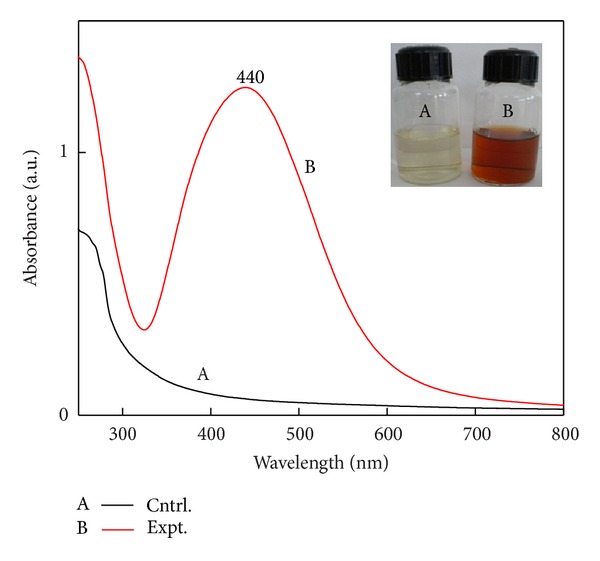
UV-Visible extinction spectra of mycosynthesized spherical SNPs colloid. Inset figure: colour change of filtrate from pale yellow (control) (A) to brown (treated) after synthesis of SNPs (B).

Appearance of dark-brown colour of fungal cell filtrate from pale yellow due to excitation of surface plasmon after treatment with silver nitrate indicates the formation of SNPs (Figure 1 inset). Synthesis of SNPs exhibits strong absorption in the visible range due to the local surface plasmon resonance. UV-Vis spectra of the samples were recorded (Figure 1). Synthesis of SNPs showed absorbance peak around 420 nm, which is specific for the SNPs. There was a single peak indicating synthesis of spherical nanoparticles. It is well known that there is a very close relationship between the UV-Vis absorbance spectrum and size and shape of SNPs. With the increase in the particle size, the optical absorption spectra of metal nanoparticles that are dominated by surface plasmon resonances (SPR) shift towards longer wavelengths (redshift) [20]. Small blueshift or red shift in the wavelength of the absorbance peak could be related to obtaining SNPs in different shape and size.

**Figure 2:**
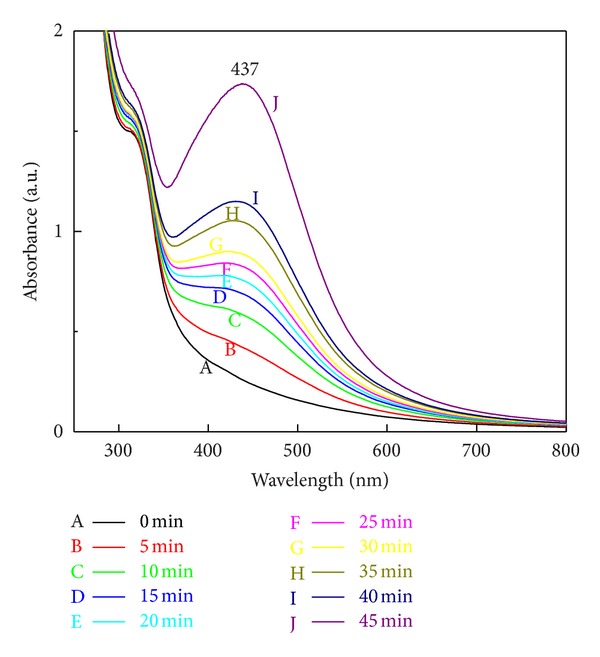
UV-Visible extinction spectra of SNPs recorded after regular time intervals of 5 min up to 45 min; synthesis of SNPs is the function of time.

To study the influence of the reaction time, we measured UV-Vis spectra of the reacting sample at regular time intervals of 5 min to 45 min. Initially, the colour of the filtrate was pale-yellow, and as time passes, colour changes to brown followed by dark-brown with increase in the absorbance, which indicates the continuous synthesis of SNPs in filtrate (Figure 2). 

### 3.3. Effect of Different Media

**Figure 3:**
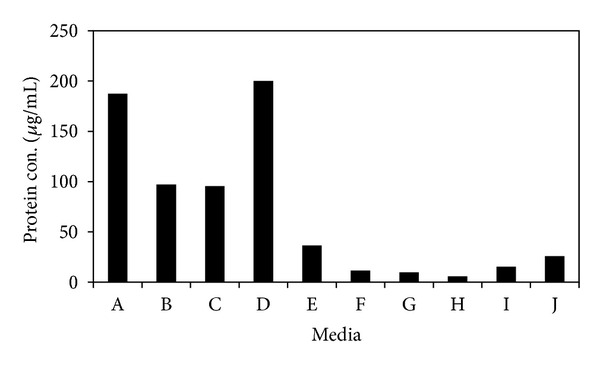
Histogram of protein concentration in different media—A: MGYP broth, B: PDB, C: lipase production medium, D: protease production medium, E: sucrose peptone yeast broth, F: gluten glucose medium, G: richard's medium, H: czapek dox medium, I: glucose peptone yeast broth, and J: sabouraud broth: Highest protein concentration is found in MGYP (A) and protease production medium (D).

**Figure 4:**
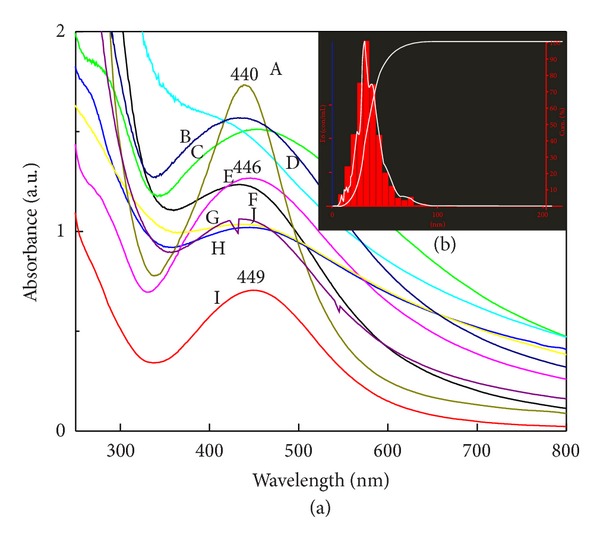
UV-Vis extinction spectroscopy of SNPs synthesized by growing fungus on different culture media—A: MGYP broth, B: PDB, C: lipase production medium, D: protease production medium, E. Sucrose peptone yeast broth, F: gluten glucose medium, G: Richard's medium, H: czapek dox medium, I: glucose peptone yeast broth, and J: sabouraud broth. (Figure 4(a) spectra A) Fungus grown on MGYP medium shows maximum synthesis with symmetry in spectrum. (Figure 4(b) inset) Analysis of sizes distribution of SNPs prepared from fungus that grew on MGYP medium by NTA.

To evaluate the effect of different media for enzyme secretion and their effect on synthesis of SNPs, we grew fungus on previously mentioned different media. The fungal biomass was grown on ten different media, mentioned previously, for 7 days. Mycelia were harvested by filtration through Whatman filter paper no. 42 and suspended in autoclaved distilled water for 24 hrs. The cell filtrate was used for protein estimation and synthesis of SNPs. The highest protein concentration was recorded in protease production medium followed by MGYP (Figure 3). Sample with high absorption intensity due to high levels of reduced silver ions or smooth curves due to uniform size distribution was obtained (Figure 4). Symmetry in graph indicates the monodispersity and stability in synthesized nanoparticles (Figure 4(b)). Among different fungal media screened, MGYP medium showed maximum surface plasmon intensity and symmetry in spectrum with peak at 440 nm followed by glucose peptone yeast broth peak at 446 nm. Sucrose peptone yeast broth showed the minimum absorbance intensity, but symmetry in spectrum indicates the monodispersity of SNPs, whereas lipase production medium, gluten glucose broth, Sabouraud broth and potato dextrose broth showed the broad peak. Czapek and Richard's broth gave the lowest surface plasmon intensity and asymmetry in graph (Figure 4(a)).

### 3.4. Effect of Different pH

**Figure 5:**
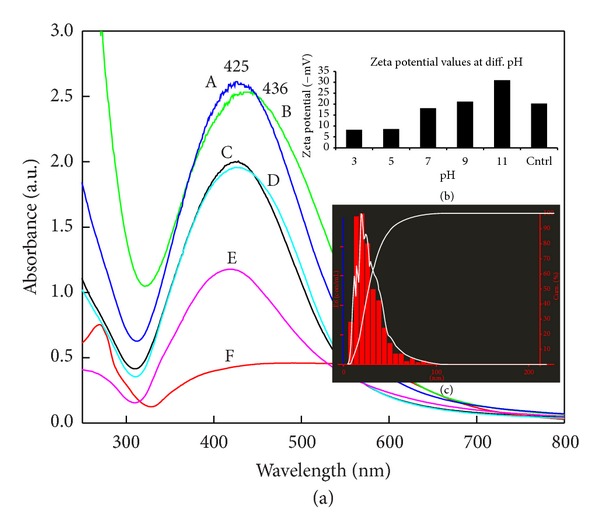
(a) UV-Vis extinction spectroscopy with the effect of pH on synthesis of SNPs. A. pH 9, B: pH 11, C: pH 7, D: control, E: pH 5, and F: pH 3. Spectra A: pH 9 and B: pH 11, show maximum synthesis of SNPs with symmetry in graph. (Figure 5(b) inset) Zeta potential values of SNPs synthesized at different pH (range of acid to alkali): pH 11, maximum zeta potential value is responsible for the stability of SNPs. Monodispersity ((c) inset): particle size distribution by NTA of pH 11.

The alkaline pH 9 and pH 11 showed the maximum synthesis of nanoparticles while in acidic pH 3 and pH 5 aggregates were observed. At pH 7, there was less synthesis of SNPs as compared to alkaline pH (Figure 5(a)). It was observed that pH of the filtrate was lowered after the synthesis of SNPs. There was no evidence of flocculation of particles at alkaline pH which indicates that the nanoparticles were monodispersed and stable at alkaline pH, whereas at acidic pH aggregates were formed within few days. NTA showed the larger particles at pH 3 and pH 5 due to aggregates formation, while at alkaline pH 9 and pH 11 monodispersed stable SNPs of mode size 30 nm and 24 nm (Figure 5(c) inset) were found, respectively. Zeta potential value of SNPs at alkaline pH was found to be quite higher, and particles were relatively stable due to the electrostatic repulsion which might be due to the adsorption of OH^−^ on SNPs, while at acidic pH aggregates were formed due to unavailability of OH^−^ ions and show low value of zeta potential. Zeta potential of SNPs increases with increasing pH (Figure 5(b) inset).

### 3.5. Effect of Different Temperatures on Extracellular Protein Secretion and Rate of Synthesis of SNPs

**Figure 6:**
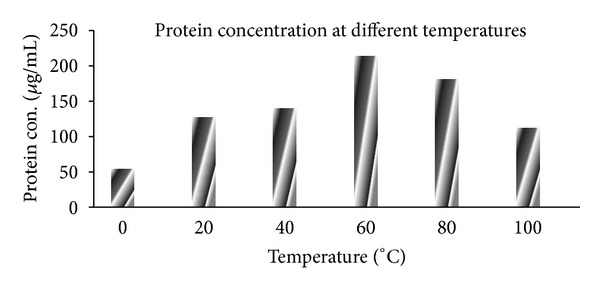
Effect of temperature on extracellular protein secretion by fungal biomass: biomass kept at 60°C shows maximum protein secretion.

**Figure 7:**
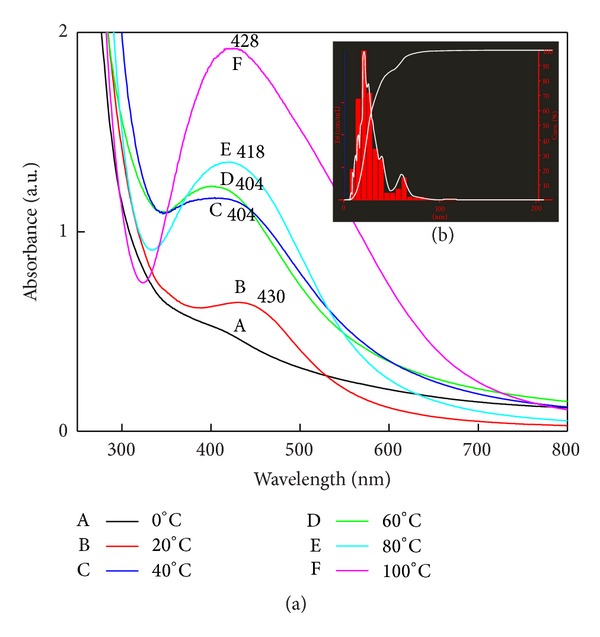
Effect of temperature on size of SNPs: (a) UV-Vis spectral analysis shows reduction of SNPs. A: 0°C, B: 20°C, C: 40°C, D: 60°C, E: 80°C, and F: 100°C. Spectrum C and spectrum D have the blue shift which indicates the small size of synthesized SNPs. Spectrum F asymmetric spectra indicate the aggregation of particles at high temperature. ((b) inset) Particle size distribution by NTA of sample prepared at 60°C.

In order to study the effect of temperature on extracellular protein secretion by fungi, biomass was exposed to different temperatures, namely, 0°C 20°C, 40°C, 60°C, 80°C, and 100°C (Figure 6). Maximum protein secretion was found at 60°C to 80°C, which may be due to the heat shock. The temperature is one of the important factors in any chemical and biological reaction as it affects the rate of reaction. The fungal filtrate treated with 1 mM AgNO_3_ were kept at different temperature same as above with respective control, to study the effect of different temperature on the rate of synthesis of SNPs, polydispersity and impact on nanoparticles size. Gradual increase in rate of synthesis of SNPs and surface plasmon absorbance was observed with increase in temperature (Figure 7(a)). The increase in the absorbance indicates the increase in the number of nanoparticles or increase in size of individual SNPs. For 40°C and 60°C, the surface plasmon resonance band showed high intensity with dark brown colour of the filtrate. Particles size distribution at 40°C and 60°C was recorded with a mode of 21 and 40 nm, respectively (Figure 7(b) inset). 

### 3.6. Effect of Different Light Intensities

**Figure 8:**
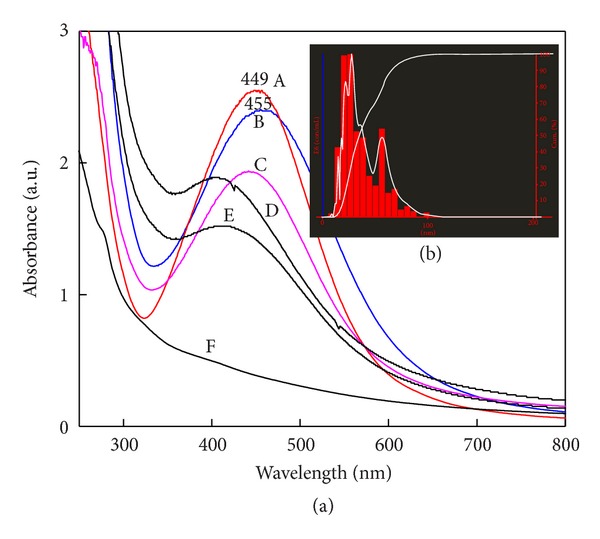
Effect of light intensity on SNPs synthesis. A: 750 × 100 lux (sunlight), B: 141.3 lux (40 Watt yellow light), C: 190.7 lux (40 Watt fluorescence light), D: in dark, E: 93.1 lux (UV light), and F: 15.5 lux (15 Watt light). (a) Sunlight (spectrum A) shows maximum surface plasmon absorbance which indicates the maximum synthesis (within 10 min) and monodispersity of SNPs followed by 141.3 lux light intensity (spectrum B). In dark, more than 48 hrs were required for complete reduction of silver ions. Particle size distribution by NTA of sample prepared in sunlight ((b) inset).

Although photochemical synthesis of metal nanoparticles dates back to a discovery in the 18th century, it is well known that certain silver salts darken by the irradiation of light. Later, the reaction “photolysis of silver salts”  was used for photographic film emersions and provided a dramatic development in photographic techniques. Nowadays, a variety of photoinduced synthetic methods for metal nanoparticle synthesis and nanostructure has been developed to obtain well-defined monometallic and bimetallic nanoparticles and composite materials. Effect of different light intensities on the rate and size of SNPs (Figure 8) provides evidence that sunlight showed complete reduction of silver ions to SNPs within 10 min with peak at 449 nm, maximum absorbance and symmetry (Figure 8(a)). Nanoparticles size distribution by NTA showed mean size of 42 nm and mode of 36 nm (Figure 8(b) inset). Light intensity, 190.7 and 141.3 lux also demonstrated symmetric graph with narrow size, but it requires more time for complete reduction of silver ions. SNPs synthesized at 141.3 lux showed absorbance peak at 455 nm with red shift in spectra. In support of UV-Vis spectra, NTA size distribution analysis also demonstrated SNPs with slightly bigger size of mode 37 nm. In dark the synthesis of SNPs was obtained in 48 hrs. At 15.5 lux, even after 72 hrs, there was no evidence of synthesis. The absorbance increases with increasing light intensity. Interestingly, synthesis of SNPs was also recorded in UV light after 24 hrs with peak at 407 nm, that is, with smaller size SNPs.

### 3.7. Effect of Different Quantity of Biomass Suspended in Distilled Water

**Figure 9:**
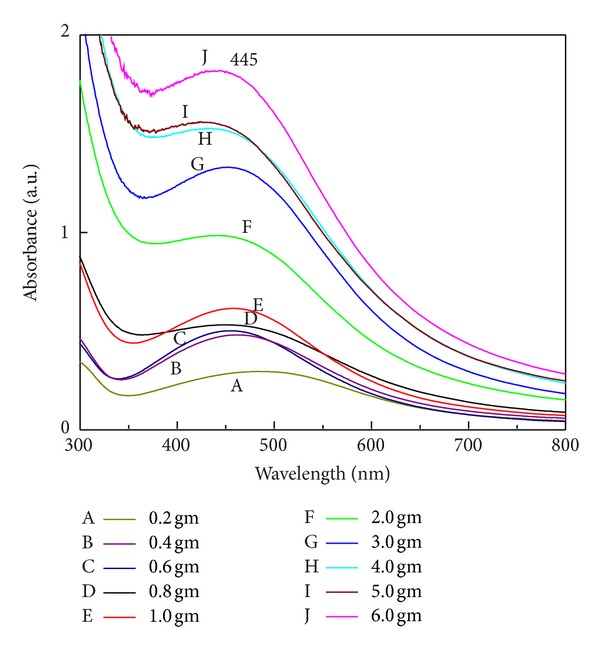
Effect of quantity of biomass (g/100 mL) on synthesis of SNPs. UV-Vis extinction spectra show increase in absorbance with increase in biomass quantity. Quantity of biomass is proportional to synthesis of SNPs.

By employing the variable amount of biomass, the effect of biomass amount on synthesis of SNPs was studied by UV-Vis spectral analysis and provided evidence that increase in quantity of biomass from 0.2 g to 6.0 g suspended in 100 mL of sterilized distilled water increases the synthesis of SNPs with steady peak (Figure 9). The amount of biomass plays a key role in synthesis or complete reduction of Ag^+^ to Ag^0^. 

### 3.8. Effect of Different Volume of Filtrate and Different Concentrations of Silver Nitrate

**Figure 10:**
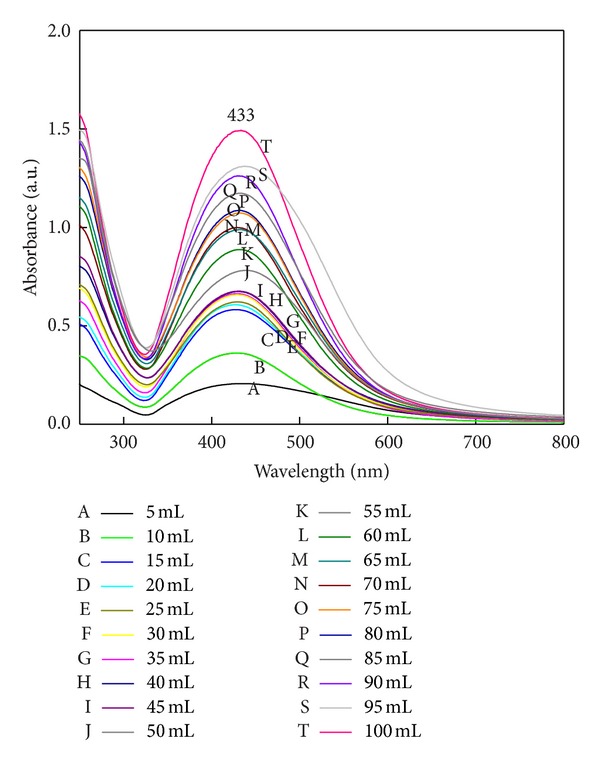
Effect of filtrate volume on synthesis of SNPs (1 mM AgNO_3_). 100% reduction of silver ions takes place in 100 mL of filtrate.

The effect of quantity of fungal filtrate on rate of SNPs synthesis was studied at fixed concentration of silver nitrate (1 mM) on different quantity of filtrate 5 mL, 10 mL, and 20 mL up to 100 mL (total volume 100 mL) and different concentration of silver nitrate on fixed quantity of fungal filtrate. UV-Vis spectra showed increase in intensity of surface plasmon absorbance with increasing filtrate volume (Figure 10). In 100 mL of filtrate (100% concentrated) maximum absorbance indicates the complete reduction of silver ions and rapid synthesis of SNPs. The rate of synthesis increased with an increased filtrate volume.

**Figure 11:**
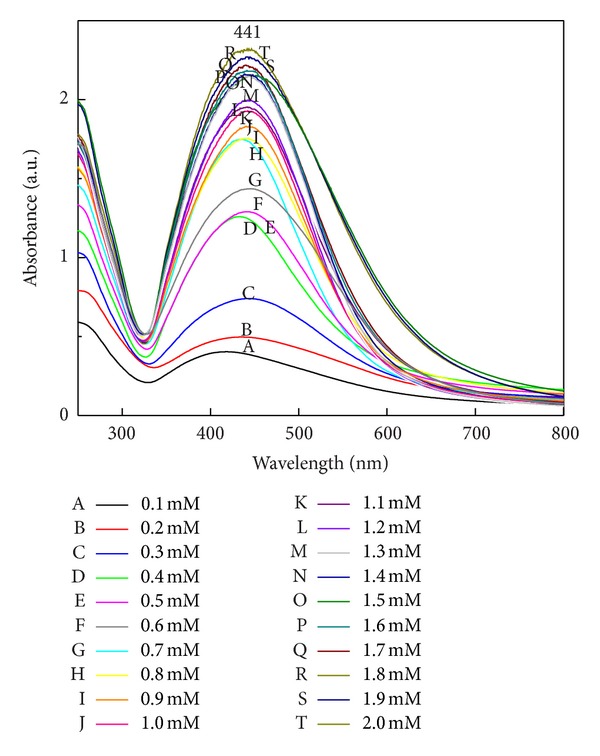
Effect of salt concentration on synthesis of SNPs. UV-Vis extinction spectra show maximum surface plasmon absorbance in 2 mM (T) concentration of SNPs. Synthesis of SNPs is proportional to salt concentration (treatment of 1.5 mM AgNO_3_ gives maximum synthesis of SNPs as compared with 2 mM AgNO_3_).

The optimum concentration of silver nitrate (1 mM) is used for the synthesis of SNPs [21]. Here we tried for maximum synthesis of SNPs with increased concentration of silver nitrate from 0.1 mM, 0.2 mM, 0.3 mM, and 0.4 up to 2 mM. Surface plasmon absorbance increases with increased salt concentration (Figure 11). Maximum synthesis takes place at 1.5 mM. Further increase in SNPs concentration was observed with little difference in surface plasmon absorbance. 1.5–1.8 mM silver nitrate concentration is suitable for maximum synthesis.

### 3.9. FTIR Analysis of SNPs Synthesized under Optimized Conditions

**Figure 12:**
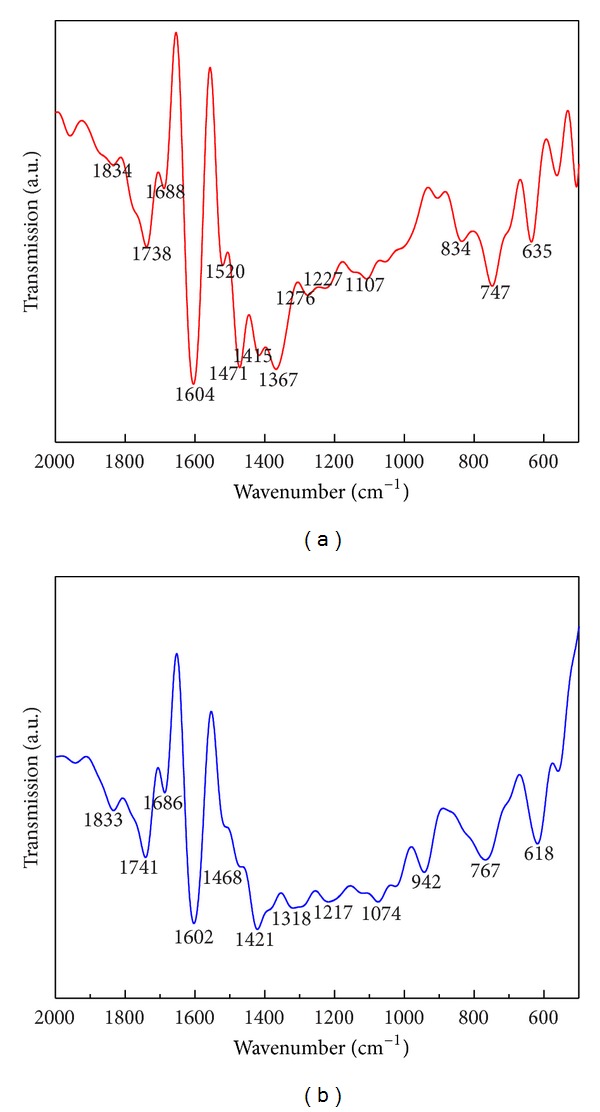
FTIR spectra recorded from SNPs powder synthesized using *F. oxysporum* by applying optimized conditions (spectra (a): control, (b): experimental).

FTIR measurements of the samples of dried powder were carried out to identify the probable interactions between silver and bioactive molecules, which may be responsible for synthesis and stabilization (capping material) of SNPs. In the present spectra, peaks at 1834, 1688, 1604, 1471, 1227, 1107, and 635 (Figure 12, spectra (a)) are stretched to 1833, 1686, 1602, 1468, 1217, 1074, and 618, respectively (Figure 12 spectra (b)). It indicates the involvement of different functional groups of amino acid in the synthesis of SNPs, which gives the specific signature spectra in electromagnetic spectrum. 

### 3.10. TEM Analysis of SNPS Synthesized under Optimized Conditions

**Figure 13:**
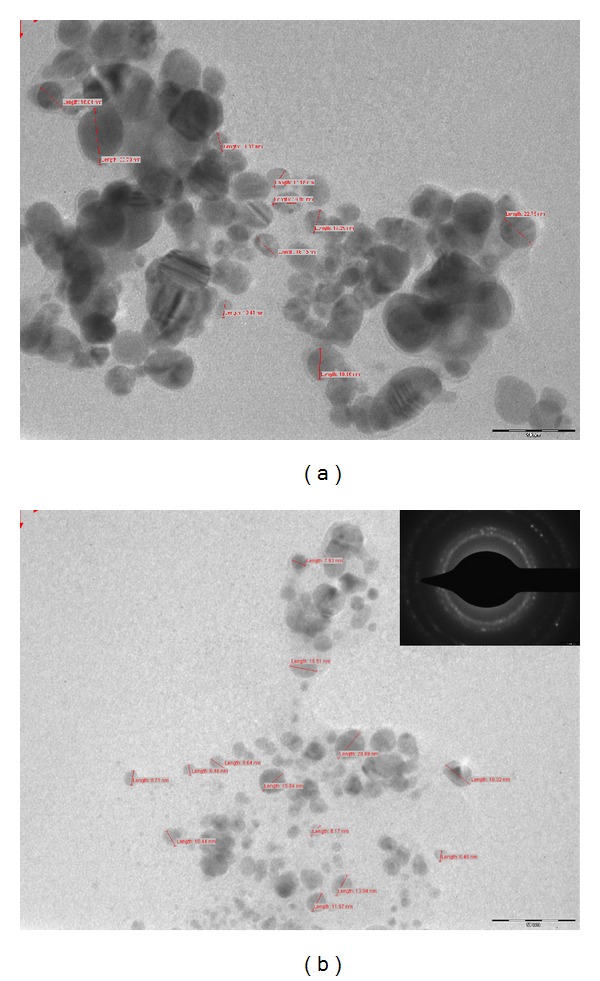
(a) TEM micrograph of SNPs synthesized using *F. oxysporum* without applying optimized conditions showed SNPs in range 10–40 nm (scale bar—50 nm), (b) SNPs of size 10–20 nm synthesized by applying optimized condition (scale bar—50 nm) (inset: diffraction spot reveals the polycrystalline nature of silver).

The sizes of SNPs synthesized with normal conditions are in the range from 10 to 40 nm and large distribution of sizes is in the range of 15–35 nm with yield of 120.30 mg/lit (Figure 13(a)). On the other hand, SNPs sizes in the range of 10–20 nm were more abundant, although tiny SNPs (4–10 nm) were also synthesized by applying optimized conditions (Figure 13(b)) and yield of SNPs was 197.20 mg/lit. Additionally, crystalline nature of SNPs was confirmed by TEM having selected area electron diffraction (SAED) attachment. The SAED patterns (Figure 13(b) inset) from the samples revealed well-defined diffraction spots in the form of rings, which indicate polycrystalline nature of silver. 

## 4. Discussion

There are different physical and chemical routes to synthesize the SNPs, but the biological route is more important due to easy accessibility. In fact, in order to increase the yield of SNPs, we studied the effect of different physicocultural parameters. 

In case of influence of reaction time, it was observed from spectra that surface plasmon resonance band occurs at 437 nm and this absorption steadily increases at fixed wavelength as a function of time of reaction. Figure 2 shows that the intensity of the absorbance peak increases with the increase in reaction time, which indicates the continued reduction of the silver ions. Increase of the absorbance with the reaction time indicates that the concentration of SNPs increases. After 45 min also peak was steadily observed at 437 nm, and there is no further increase in the absorbance, indicating the complete reduction of the silver ions. 

It is well known that in different culture media conditions and compositions microbial cell responds differently and secretes different metabolites and different kinds of proteins. Also, we know that the biological synthesis of SNPs is enzymes catalyzed reaction [22]. In case of maximum production of SNPs, fungi should secrete specific enzymes or metabolites which are responsible for reduction of silver ions, high growth rate and low cost requirement for production procedure. In the present study, MGYP medium may promote the extracellular nitrate reductase secretion and hence enhance the synthesis of SNPs (Figure 3). The symmetric graph indicates the optimum conversion of silver ions to SNPs (Figure 4(a)), which may be due to the secretion of nitrate reductase, an enzyme responsible for the reduction of silver ions [22]. The particles size distribution of respective SNPs showed the mode of 30 nm (Figure 4(b) inset). The larger size of the nanoparticles predicted by NTA may be due to the capping of SNPs by different extracellularly secreted proteins. Proteins have multiple effects on the dispersion, including potential screening of the surface charges that help to maintain the repulsion between the particles, or bridging type interactions [23].

In case of effect of pH study, pH 9 and pH 11 showed the maximum absorbance and peak at 425 nm and 436 nm, respectively, which showed the blueshift in spectra with increasing pH (Figure 5(a) spectra A and B). OH^−^ ions are nucleophiles which play crucial role in maintaining the stability of SNPs by adsorbing on it and in synthesis of smaller size SNPs by providing electrons for reduction of silver ions. More nucleation regions are formed due to the availability of OH^−^ ions which help in preventing the aggregates are formation through adsorbing on nanocrystals and maintains the smaller size of SNPs [24]. NTA results showed (Figure 5(c)) similarity with Chen and Carroll [25] findings that at alkaline pH SNPs are stable and aggregates formed at lower pH. It indicates that, by controlling the pH of SNPs synthesis, it is easy to control the size of SNPs. Nayak et al. [26] hypothesized that the proton concentration affects conformational changes in the nitrate reducing enzymes present in the fungal filtrate, which may change the morphology and size of the SNPs. Deepak et al. [27] stated that when the condition of the SNPs mycofabrication is alkaline, the synthesis will be faster than in acidic conditions. In other words, synthesis enhances as the pH increases towards alkaline region. In alkaline conditions there was no need of agitation of the mixture for the formation of SNPs and all the silver ions supplied were converted to SNPs within 10 min. The proteins involved in the synthesis may bind with silver at thiol regions (–SH) forming an S–Ag bond, a clear indication of which aids the conversion of Ag^+^ to Ag^0^. In addition, the alkaline ion (–OH) is very much required for the reduction of metal ions. Moreover, under alkaline conditions, the ability of the 243 enzymes responsible (not only nitrate reductase) for the synthesis of SNPs increases [28]. The present findings corroborate with Sintubin et al. [29] who proposed the mechanism for the synthesis of SNPs by using lactic acid bacteria. The authors reported that whenever pH increases, more competition occurs between protons and metal ions for negatively charged binding sites; therefore, a better synthesis at higher or alkaline pH as compared with lower or acidic pH has been recorded. 

While studying temperature effect it was found that as the temperature increases the absorbance was also increase (Figure 7). Complete reduction of silver ions to SNPs is corroborated with the result of Darroudi et al. [30], which shows the increase in SNPs synthesis with increasing temperature. At 20°C, peak arises at 430 nm, whereas at 40°C and 60°C peak is observed at 404 nm; there was blue shift that indicates the monodispersed and reduction in size of SNPs (Figure 7(a)). Again, red shift and asymmetry with broad peak width were observed at 80°C and 100°C which start off aggregation of SNPs due to denaturation of proteins capping at high temperature, which leads to the variations in the nucleation of Ag^+^ species and growth rate. At elevated temperature, increased polydispersity and formation of nanoparticles at a faster rate is inferred from the UV-VIS absorbance intensity in Figure 7(a). At 0°C, no peak was observed, which means that SNPs synthesis did not take place. Temperature range of 40–60°C is suitable for stable synthesis of SNPs with short time period. NTA analysis data of SNPs synthesized at different temperature support the UV-Vis spectral analysis (Figure 7(b)). NTA of 20°C showed the minimum mode size of 21 nm but takes the maximum time to complete the reduction process. At lower temperature, reduction reaction could not finish completely within short time so that less SNPs were formed. At 100°C, the total reduction of silver ions could take place within 30–40 minutes, but, after completion of reaction, formation of aggregates was initiated within few days. Sarkar et al. [31] reported that, with increase of temperature, the kinetic energy of the SNPs in the solution also increases; as a result, the collision frequency between the particles also rises, and this leads to the higher rate of agglomeration. Similarly, NTA also showed the particles with large size due to change in surface potential. Surface potential of SNPs is inversely proportional to temperature which leads to the formation of aggregates [32]. They demonstrated the particles growth rate over a range of ionic strengths and reaction temperature in which particles interact via electrostatic and Van der Waals forces. At 40°C, SNPs were synthesized within 5-6 hours, whereas at 60°C in 3 hours. Lee and Chen [33] also demonstrated that the photomediated SNPs shape conversion process gets fast at higher temperature and slowed at lower temperature. Consequently, it can be concluded that the rate of particles formation and the size of particles can be controlled by temperature.

There are very few reports on photomediated biological synthesis of SNPs. UV photoreduction of silver ions through binding of carboxylic acid was reported [34]. Reduction of silver ions is the synergistic effect of fungal filtrate containing protein and ambient light while the control without fungal filtrate did not show any synthesis of SNPs. Visible light plays a significant role in complete reduction of silver ions with fast rate. Rapid extracellular synthesis in sunlight may be due to the photosensitization of aromatic compounds in the fungal filtrate, in which free electron of aromatic compound would be utilized by silver ion to get reduced to nanoparticles. Silver ion reduction through carboxylic acid functional groups of yeast surface peptide in the presence of ambient visible light was reported by Nam et al. [35]. Similarly, culture supernatant of *Klebsiella pneumoniae* results in the SNPs synthesis under the irradiation of visible light [36]. 

**Figure 14:**
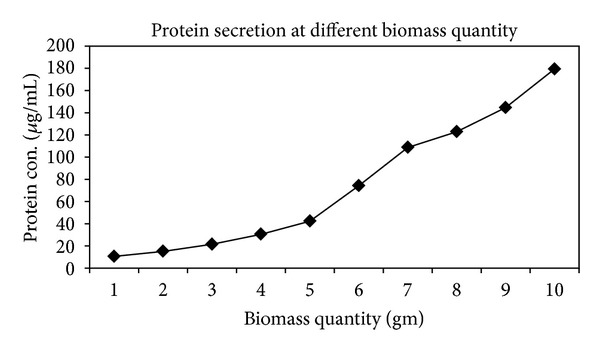
Effect of quantity of biomass on protein secretion. As the quantity increases, protein secretion also increases (protein estimation by Folin-Lowry's method).

The number of spores inoculated in liquid culture medium also affects the synthesis of SNPs (Figure 9). The quantity of biomass plays a key role in synthesis and complete reduction of Ag^+^ to Ag^0^. Protein estimation demonstrated the increase in protein concentration as quantity of fungal biomass increases (Figure 14). Here, we tried to get more extracellular proteins and other biomolecules, which help in maximum synthesis of SNPs.

There are several reports in the literature which provide evidence for involvement of an enzyme in the synthesis of SNPs by the fungal system or that mycofabrication of SNPs is an enzyme catalyzed reaction [22, 37, 38]. There are two queries regarding the use of silver nitrate concentration for the mycofabrication of SNPs by fungal system. Firstly, for enzyme catalyzed reaction, the effect of substrate concentration (silver nitrate) is a key parameter that affects the process of nanoparticle synthesis. Very few reports are available about the study of different substrate concentration, and their effect on mycofabrication process of SNPs. Secondly, the synthesis of SNPs by using fungal system was reported by several researchers [11, 16, 38–42]. They have reported the synthesis of SNPs by using 1 mM silver nitrate concentration, but there is no explanation why only 1 mM silver nitrate is used and not less than 1 mM. To address the two questions mentioned previously, the effect of quantity of fungal filtrate on rate of SNPs synthesis was studied. In case of effect of filtrate volume on synthesis of SNPs it was observed that the rate of synthesis increased with an increased filtrate volume. In case of plant-mediated synthesis of SNPs, and gold nanoparticles 5% leaf broth is responsible for 100% conversion of respective ions [43, 44]; however, in case of fungi 100% fungal, filtrate showed maximum SNPs synthesis. Usually, the optimum concentration of silver nitrate (1 mM) is used for the synthesis of SNPs [21]. Maximum synthesis takes place at 1.5 mM. Further increase in SNPs concentration was observed with little difference in surface plasmon absorbance. 1.5–1.8 mM silver nitrate concentration is suitable for maximum synthesis (Figure 11).

FTIR spectroscopy gives the idea about the capping and interactions of protein with silver ions which is responsible for stability of SNPs. Among them, peak around 1025 cm^−1^ can be assigned as absorption peak of –C–O–C– or –C–O–, and peak at 1630 cm^−1^ is produced with the stretch vibration of –C=C– and is assigned to the amide I bonds of proteins [45]. The stretching of peaks revealed the different functional groups like C–N, C–O–C, amide linkages, and –COO– which are present between amino acid residues in protein and are involved in the synthesis of SNPs. In our study, we obtained peaks at 1686 stretching of 1688 and 1318 which may be produced due to the C–C and C–N stretching indicating the protein capping on the SNPs. The peaks at 1074 cm^−1^ correspond to C–N stretching vibration of aliphatic amines or to alcohols/phenols, representing the presence of polyphenols [43]. The peak at 1452 cm^−1^ may be assigned to symmetric stretching vibrations of –COO– (carboxylate ion) groups of amino acid residues with free carboxylate groups in the protein [46] while we obtained peak at 1468 stretching of 1471 which is around 1452 cm^−1^. Peaks at 1741, 1602, 1468, 1217, and 1074 were produced due to carbonyl stretching vibration in ketones, aldehydes, and carboxylic acid, stretching vibration of C=C, symmetric stretching vibration of –COO–, and amide III bond and C–N stretching vibration of aliphatic amines, respectively; those are derived from heterocyclic compounds like proteins, which are present in the fungal extract and are the capping ligands of the nanoparticles [45]. FTIR spectra (Figure 12) clearly indicate that the biomolecules especially proteins present in filtrate are responsible for synthesis and stabilization of SNPs in colloidal form.

SNPs were synthesized by collectively applying the previous all optimized conditions. All the SNPs were spherical in shape and showed noticeable variation in size in comparison with general synthesizing process. TEM images (Figure 13(b)) revealed smaller and maximum synthesis of stable SNPs without significant agglomeration at 60°C and at alkaline pH. 

### 4.1. Probable Mechanism for the Synthesis of SNPs

#### 4.1.1. Photoreduction of Silver Ions to SNPs

Several studies showed the involvement of nitrate reductase enzyme and different proteins in the synthesis of SNPs [22, 37, 47]. Here, experimentation also demonstrated that, without fungal cell filtrate synthesis of SNPs did not occur in sunlight. In addition, the rapid synthesis of SNPs was observed in presence of sunlight and at high temperature. However, the exact mechanism of SNPs in light has not been demonstrated. Nam et al. [35] reported that the silver ions are reduced through carboxylic acid functional groups of yeast surface peptide in the presence of ambient visible light. Callegari et al. [48] demonstrated the need of dissolved oxygen in the photoconversion of SNPs. Similarly, the UV photoreduction of silver ions through binding of carboxylic acid was reported [34]. In our photo reduction study, there may be photosensitization of aromatic amino acids of filtrate protein, which may help in reduction of silver ions by providing electrons as well as silver ions to catalyze the amine oxidation. In case of high intensity light, heat generates, which may also accelerate the rate of synthesis of SNPs, or photolysis of silver ions may take place. 

#### 4.1.2. Free Amino Acids Responsible for Synthesis of SNPs

In the present study, we found that the SNPs were synthesized even after the heat denaturation of fungal filtrate protein. The pale-yellow colour of fungal cell filtrate was changed to dark brown during the boiling of reaction mixture containing fungal filtrate and silver nitrate. The UV-Vis spectra showed the peak at 428 nm, which confirmed the synthesis of SNPs. In another experiment, the heat denatured fungal filtrate after treatment with 1 mM silver nitrate also demonstrated the synthesis of SNPs in bright sunlight. It suggests that synthesis of SNPs was due to transfer of electrons from free amino acids to silver ions. Moreover, the Ninhydrin test confirmed the presence of free amino acids in heat denatured fungal filtrate, which may be responsible for the synthesis of SNPs. In biosynthesis of SNPs by *Chlorella vulgaris* extracts, Xie et al. [49] reported the involvement of hydroxyl groups of tyrosine residues and carboxyl groups of aspartate and glutamate residues in the reduction of silver ions and anisotropic growth of SNPs.

## 5. Conclusions

On combining all optimized conditions, ecofriendly and inexpensive method has been developed for the rapid and large scale synthesis of SNPs. However, so far, there is no report on the study of effect of all these cultural and physical conditions on biological synthesis of SNP. Cultural (culture medium, quantity of biomass, filtrate volume, and salt concentration) and physical conditions (pH, temperature, and light intensity) have been found to affect the maximum yield, rate of synthesis, and size of SNPs. In sunlight, synthesis of SNPs takes place within few minutes. Exploitation of natural energy source for synthesis of SNPs can overcome the problem of energy consumption as well as rate of synthesis. In combination with all desired conditions, SNPs with uniform size distribution of 10–20 nm with stability and promising increase in yield were obtained. The study revealed that the synthesis of SNPs may take place due to amino acids. The optimization of the parameters would lead to the rapid and large scale production of SNPs at industrial level, which may be used as novel antimicrobials against multidrug resistant microorganisms.

## References

[1] Rai M, Ingle A (2012). Role of nanotechnology in agriculture with special reference to management of insect pests. *Applied Microbiology and Biotechnology*.

[2] Hristozov D, Malsch I (2009). Hazards and risks of engineered nanoparticles for the environment and human health. *Sustainability*.

[3] Garcia-Parajo MF (2012). The role of nanophotonics in regenerative medicine. *Methods in Molecular Biology*.

[4] Rai M, Yadav A, Gade A (2009). Silver nanoparticles as a new generation of antimicrobials. *Biotechnology Advances*.

[5] Wei X, Luo M, Li W (2012). Synthesis of silver nanoparticles by solar irradiation of cell-free *Bacillus amyloliquefaciens* extracts and AgNO_3_. *Bioresource Technology*.

[6] Seshadri S, Saranya K, Kowshik M (2011). Green synthesis of lead sulfide nanoparticles by the lead resistant marine yeast, *Rhodosporidium diobovatum*. *Biotechnology Progress*.

[7] Parameswari E, Lakshmanan A, Thilagavathi T (2010). Biosorption and metal tolerance potential of filamentous fungi isolated from metal polluted ecosystem. *Electronic Journal of Environmental, Agricultural and Food Chemistry*.

[8] Rai M, Yadav A, Bridge P, Gade A, Rai M, Bridge PD (2009). Myconanotechnology: a new and emerging science. *Applied Mycology*.

[9] Mishra A, Tripathy SK, Yun S (2011). Bio-synthesis of gold and silver nanoparticles from *Candida guilliermondii* and their antimicrobial effect against pathogenic bacteria. *Journal of Nanoscience and Nanotechnology*.

[10] Soni N, Prakash S (2012). Efficacy of fungus mediated silver and gold nanoparticles against *Aedes aegypti* larvae. *Parasitology Research*.

[11] Vahabi K, Mansoori GA, Karimi S (2011). Biosynthesis of silver nanoparticles by fungus *Trichoderma Reesei* (a route for large-scale production of AgNPs). *Insciences Journal*.

[12] Chladek G, Mertas A, Barszczewska-Rybarek I (2011). Antifungal activity of denture soft lining material modified by silver nanoparticles: a pilot study. *International Journal of Molecular Sciences*.

[13] Knetsch MLW, Koole LH (2011). New strategies in the development of antimicrobial coatings: the example of increasing usage of silver and silver nanoparticles. *Polymers*.

[14] Kokura S, Handa O, Takagi T, Ishikawa T, Naito Y, Yoshikawa T (2010). Silver nanoparticles as a safe preservative for use in cosmetics. *Nanomedicine*.

[15] Sheng Z, Liu Y (2011). Effects of silver nanoparticles on wastewater biofilms. *Water Research*.

[16] Birla SS, Tiwari VV, Gade AK, Ingle AP, Yadav AP, Rai MK (2009). Fabrication of silver nanoparticles by *Phoma glomerata* and its combined effect against *Escherichia coli, Pseudomonas aeruginosa* and *Staphylococcus aureus*. *Letters in Applied Microbiology*.

[17] Lara HH, Garza-Treviño EN, Ixtepan-Turrent L, Singh DK (2011). Silver nanoparticles are broad-spectrum bactericidal and virucidal compounds. *Journal of Nanobiotechnology*.

[18] Seifert K (1996). *Fusarium Interactive Key*.

[19] Lesliey BA, Summerell JF *The Fusarium Laboratory Manual*.

[20] Shafeev GA, Freysz E, Bozon-Verduraz F (2004). Self-influence of a femtosecond laser beam upon ablation of Ag in liquids. *Applied Physics A*.

[21] Ahmad A, Mukherjee P, Senapati S (2003). Extracellular biosynthesis of silver nanoparticles using the fungus *Fusarium oxysporum*. *Colloids and Surfaces B*.

[22] Kumar SA, Abyaneh MK, Gosavi SW (2007). Nitrate reductase-mediated synthesis of silver nanoparticles from AgNO_3_. *Biotechnology Letters*.

[23] Montes-Burgos I, Salvati A, Lynch I, Dawson K Characterization techniques for nanoparticle dispersion.

[24] Gurunathan S, Kalishwaralal K, Vaidyanathan R (2009). Biosynthesis, purification and characterization of silver nanoparticles using *Escherichia coli*. *Colloids and Surfaces B*.

[25] Chen S, Carroll DL (2004). Silver nanoplates: size control in two dimensions and formation mechanisms. *Journal of Physical Chemistry B*.

[26] Nayak RR, Pradhan N, Behera D (2011). Green synthesis of silver nanoparticle by *Penicillium purpurogenum* NPMF: the process and optimization. *Journal of Nanoparticle Research*.

[27] Deepak V, Kalimuthu K, Sureshbabu RKP, Gurunathan S, Rai MK, Duran N (2011). Deepak et al book chapter. *Metal Nanoparticles in Microbiology*.

[28] Sanghi R, Verma P (2009). A facile green extracellular biosynthesis of CdS nanoparticles by immobilized fungus. *Chemical Engineering Journal*.

[29] Sintubin L, de Windt W, Dick J (2009). Lactic acid bacteria as reducing and capping agent for the fast and efficient production of silver nanoparticles. *Applied Microbiology and Biotechnology*.

[30] Darroudi M, Ahmad MB, Abdullah AH, Ibrahim NA (2011). Green synthesis and characterization of gelatin-based and sugar-reduced silver nanoparticles. *International Journal of Nanomedicine*.

[31] Sarkar S, Jana AD, Samanta SK, Mostafa G (2007). Facile synthesis of silver nano particles with highly efficient anti-microbial property. *Polyhedron*.

[32] van Hyning DL, Klemperer WG, Zukoski CF (2001). Silver nanoparticle formation: predictions and verification of the aggregative growth model. *Langmuir*.

[33] Lee Y, Chen S (2010). Finding a facile method to synthesize decahedral silver nanoparticles through a systematic study of temperature effect on photomediated silver nanostructure growth. *Journal of the Chinese Chemical Society*.

[34] Maillard M, Huang P, Brus L (2003). Silver nanodisk growth by surface plasmon enhanced photoreduction of adsorbed [Ag+]. *Nano Letters*.

[35] Nam KT, Lee YJ, Krauland EM, Kottmann ST, Belcher AM (2008). Peptide-mediated reduction of silver ions on engineered biological scaffolds. *ACS Nano*.

[36] Mokhtari N, Daneshpajouh S, Seyedbagheri S (2009). Biological synthesis of very small silver nanoparticles by culture supernatant of *Klebsiella pneumonia*: the effects of visible-light irradiation and the liquid mixing process. *Materials Research Bulletin*.

[37] Durán N, Marcato PD, Alves OL, de Souza GIH, Esposito E (2005). Mechanistic aspects of biosynthesis of silver nanoparticles by several *Fusarium oxysporum* strains. *Journal of Nanobiotechnology*.

[38] Ingle A, Gade A, Pierrat S, Sönnichsen C, Rai M (2008). Mycosynthesis of silver nanoparticles using the fungus *Fusarium acuminatum* and its activity against some human pathogenic bacteria. *Current Nanoscience*.

[39] Gade AK, Bonde P, Ingle AP, Marcato PD, Durán N, Rai MK (2008). Exploitation of *Aspergillus niger* for synthesis of silver nanoparticles. *Journal of Biobased Materials and Bioenergy*.

[40] Ingle A, Rai M, Gade A, Bawaskar M (2009). Fusarium solani: a novel biological agent for the extracellular synthesis of silver nanoparticles. *Journal of Nanoparticle Research*.

[41] Fayaz M, Tiwary CS, Kalaichelvan PT, Venkatesan R (2010). Blue orange light emission from biogenic synthesized silver nanoparticles using *Trichoderma viride*. *Colloids and Surfaces B*.

[42] Castro-Longoria E, Vilchis-Nestor AR, Avalos-Borja M (2011). Biosynthesis of silver, gold and bimetallic nanoparticles using the filamentous fungus *Neurospora crassa*. *Colloids and Surfaces B*.

[43] Song JY, Kim BS (2009). Rapid biological synthesis of silver nanoparticles using plant leaf extracts. *Bioprocess and Biosystems Engineering*.

[44] Song JY, Jang H, Kim BS (2009). Biological synthesis of gold nanoparticles using *Magnolia kobus* and *Diopyros kaki* leaf extracts. *Process Biochemistry*.

[45] Sastry M, Ahmad A, Khan MI, Kumar R (2003). Biosynthesis of metal nanoparticles using fungi and actinomycete. *Current Science*.

[46] Shankar SS, Rai A, Ahmad A, Sastry M (2004). Rapid synthesis of Au, Ag, and bimetallic Au core-Ag shell nanoparticles using Neem (*Azadirachta indica*) leaf broth. *Journal of Colloid and Interface Science*.

[47] Jain N, Bhargava A, Majumdar S, Tarafdar JC, Panwar J (2011). Extracellular biosynthesis and characterization of silver nanoparticles using *Aspergillus flavus* NJP08: a mechanism perspective. *Nanoscale*.

[48] Callegari A, Tonti D, Chergui M (2003). Photochemically grown silver nanoparticles with wavelength-controlled size and shape. *Nano Letters*.

[49] Xie J, Lee JY, Wang DIC, Ting YP (2007). Silver nanoplates: from biological to biomimetic synthesis. *ACS Nano*.

